# Situations in Which Oxytocin Was Administrated by Paramedics in Out-of-Hospital Births: A Retrospective Analysis over Six Years in the Polish Population

**DOI:** 10.3390/jcm13237175

**Published:** 2024-11-26

**Authors:** Hanna Wiciak, Mateusz Strózik, Adam Smereka, Tomasz Fuchs, Jacek Smereka

**Affiliations:** 1 Clinical Department of Gynecologic Surgery and Oncology, University Center of Obstetrics and Gynecology, Wroclaw Medical University, 50-367 Wroclaw, Poland; 2 Clinical Department of Obstetrics and Gynecology, University Center of Obstetrics and Gynecology, Wroclaw Medical University, 50-367 Wroclaw, Poland; m.strozik@umw.edu.pl (M.S.); tomasz.fuchs@umw.edu.pl (T.F.); 3 Department of Gastroenterology and Hepatology, Faculty of Medicine, Wroclaw Medical University, 50-367 Wroclaw, Poland; adam.smereka@umw.edu.pl; 4 Department of Emergency Medical Service, Wroclaw Medical University, 50-367 Wroclaw, Poland; jacek.smereka@umw.edu.pl

**Keywords:** oxytocin, postpartum haemorrhage, birth, ambulance, EMS, paramedic

## Abstract

**Introduction:** Postpartum haemorrhage (PPH) is a leading cause of maternal mortality worldwide, particularly in low- and middle-income countries, complicating 1% to 10% of deliveries. Despite improvement in prevention and management, variations in PPH definitions and measurement methods contribute to challenges in accurately assessing its incidence, with up to 90% of PPH-related deaths in high-income countries deemed avoidable through timely intervention. Oxytocin is the primary drug administered during labour or miscarriage, causing an increase in uterine muscle tone, which reduces bleeding and the risk of complications. The aim of the study was to assess the rate of oxytocin use by paramedics for out-of-hospital births in Poland and to verify adherence to WHO-recommended protocols for preventing postpartum haemorrhage in emergency prehospital settings. **Methods**: We conducted a cross-sectional study using data from the Polish Central System for Emergency Medical Services Missions Monitoring covering all EMS interventions nationwide from 2018 to 2023. The study included cases where oxytocin was administered during EMS interventions for pregnant women, identified through ICD-10 codes (O30–O92), with 62 verified cases meeting the inclusion criteria. **Results**: Over 6 years, oxytocin was administered in 62 cases when paramedics responded to emergencies involving pregnant women. The mean age of the patients to whom the oxytocin was administered was 29.48 years (SD = 6.25) and ranged from 15 to 43 years. **Conclusions**: Oxytocin is rarely administered by EMS teams at the prehospital stage. Oxytocin should be considered for incorporation into the set of medications that EMS teams can administer in prehospital settings. There is a need to train EMS teams in the management of pregnancy-related emergencies in accordance with the current medical guidelines.

## 1. Introduction

Postpartum haemorrhage (PPH) is an obstetric emergency that complicates 1% to 10% of all deliveries. In 2015, it was reported to be responsible for more than 80,000 maternal deaths worldwide [1,2]. However, the incidence of PPH varies significantly based on the definitions and criteria used, the methods for measuring postpartum blood loss, and the population studied. The highest incidence is reported in low- and lower-middle-income countries [3], and in those countries, it is also the leading cause of maternal mortality. In contrast, in developed countries, PPH has fallen to second or third place for direct maternal deaths, behind hypertensive and thromboembolic complications. In high-income countries, 80 to 90% of maternal deaths from PPH may be avoidable, as they often result from inadequate treatment or delays in diagnosis and management [4,5]. These findings are confirmed by confidential investigations indicating that up to 67% of deaths in the United States and 85% in France could be prevented, as they result from delayed or insufficient treatment [6,7,8].

Early postpartum haemorrhage (EPH) is typically defined as blood loss greater than or equal to 1000 mL of blood loss with hemodynamic instability, irrespective of the mode of delivery following vaginal delivery (VD) or a cesarean section (CS) within 24 h postpartum. Most women with normal haemoglobin and hematocrit can lose up to 1000 mL without a significant drop in blood pressure or an increase in their pulse rate. Late postpartum haemorrhage (LPH) occurs after 24 h following labour and complicates 0.23% of deliveries [9,10]. Accurately determining the rate of PPH is relatively difficult due to the lack of a universal definition of PPH, the inconsistent presentation of PPH worldwide, and the challenges in estimating blood loss during childbirth. Additionally, the actual risk to the patient and individual factors such as the bleeding rate, patient body size, and tolerance to blood loss are also important considerations [11].

Currently, synthetic oxytocin is the preferred uterotonic of choice because of its safety profile and clinical efficacy. According to the World Health Organisation (WHO) recommendations, the administration of oxytocin (10 international units [IU], intramuscular or intravenous) is advised to prevent PPH in all births. When vaginal birth allows for intravenous access, the slow intravenous administration of 10 IU oxytocin is preferred over intramuscular administration [12].

This study investigated oxytocin administration by Emergency Medical Services (EMS) teams in Poland. The aim of the research was to assess the rate of oxytocin use by paramedics during out-of-hospital births in Poland and to verify adherence to WHO-recommended protocols for preventing PPH in emergency prehospital settings.

## 2. Materials and Methods

We conducted a cross-sectional study using data from the National Centre for Emergency Medical Services, covering 2018–2023 and including all EMS interventions nationwide. The register maintained by the National Centre for Emergency Medical Services documents all rescue actions undertaken by Emergency Medical Services teams in Poland in a standardised scheme. The introduction of this system and scheme in 2017 enabled consistent analysis and data comparisons.

The original database, prepared by the National Centre for Emergency Medical Services, was based on ICD-10 codes ranging from O30 to O92 to isolate cases involving pregnant women. This range of codes allowed for the inclusion of obstetric care and the use of oxytocin during the second or third stage of labour in cases of advanced pregnancy. The inclusion criteria for the study were emergency team calls to the patient, regardless of the reason, and the use of oxytocin by the EMS team during the intervention. The only exclusion criterion was pregnancy below 22 weeks.

**Figure 1:**
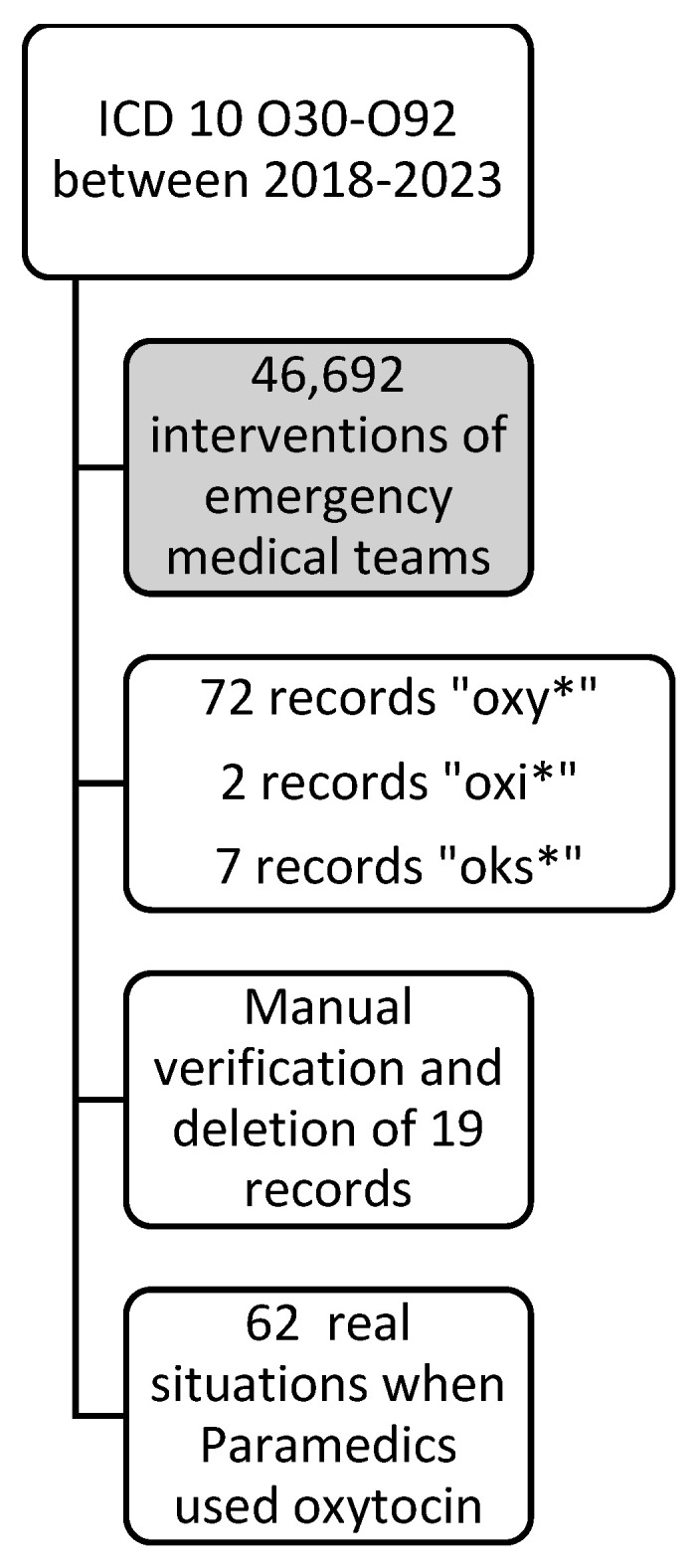
Exclusion and inclusion criteria for the study. ICD 10—International Classification of Diseases, 10th Revision; *—means any character.

The original database was searched for medications used by the emergency medical teams. Various terms that could correspond to oxytocin used during the interventions were searched. A total of 81 records were obtained, which were then manually verified. After excluding incorrect results, 62 interventions involving the administration of oxytocin were ultimately identified- data presented in Figure 1.

The assumption of normality for the t-student test was not met (the Shapiro–Wilk test was used). Medians and quartiles were reported. The U–Mann–Whitney test (Wilcoxon test for sum rank for independent samples, hereafter referred to as the M-W test) was used. The effect of the M-W test was calculated (the non-directional formula used for rank-dual correlation was proposed by Wendt and only takes positive values). The level of statistical significance was assumed to be α = 0.05. Calculations were performed manually and using Statistica (version 13.3.721.1).

The Bioethics Committee of the Wroclaw Medical University, Poland (approval No.: KBkanc.270/2024 of 15 October 2024) assessed that the study met the criteria of ethical principles in science, including the tenets of the Declaration of Helsinki. The analysed database did not include any information that could identify the patients or the EMS teams providing care during the interventions.

## 3. Results

The survey finally involved 62 EMS team interventions when paramedics administered oxytocin to pregnant patients. The average age of the patients was 29.48 years (SD = 6.25); the youngest woman was 15 years old, and the oldest was 43 years old.

Most patients were pregnant for the second time (22; 35.48%) and were giving birth for the second time (15; 24.19%). Some of the reports prepared by the EMS teams were incomplete. Therefore, in 4 (6.45%) and 19 (30.65%) cases, there was no information about which pregnancy and childbirth, respectively, it was.

The majority of the patients were in the 39th week of pregnancy. However, it is noteworthy that in as many as 10 cases (16.13%), the patient claimed that she was unaware of being pregnant. In as many as eight cases (12.90%), the EMS teams omitted the information regarding the week of pregnancy in their reports. Additionally, the cases described included 37 (59.68%) full-term pregnancies (a pregnancy that has reached 37–42 weeks of gestation) and 7 (11.29%) premature deliveries (a pregnancy that ends before 37 weeks of gestation).

In most cases (36; 58.06%), the condition of the newborn was not assessed with the use of the Apgar score by the EMS team. In one situation, the delivery was still ongoing. Among the remaining cases, 21 newborns (33.87%) were assigned a score of 10, 2 newborns (3.23%) received a score of 8, and in 2 cases (3.23%), a score of 0 was recorded.

The general and delivery-related information on the investigated interventions is provided in Table 1.

In most cases, the call to a pregnant woman where oxytocin was used was designated with the ICD-10 code O80—Single delivery (44 times; 71%). The next codes were O60—Preterm labour (7 times; 11%), O62—Prolonged labour (7 times; 11%), O63—Obstructed labour (1 time; 2%), and O72—Haemorrhage during labour and delivery (1 time; 2%); in two cases, no ICD-10 code was recorded.

Additionally, information was collected regarding the time that the EMS team needed from the moment they were called to the moment they arrived to the pregnant woman. Detailed data are presented in Table 2

Moreover, a question was posed if the response time statistically differed depending on whether a P-type (basic) or S-type (specialist) EMS team arrived to the patient. It turned out that the P-type EMS teams reached the patients statistically significantly faster than the S-type teams (Table 3).

## 4. Discussion

In 2020, WHO published their guidelines regarding the use of intravenous versus intramuscular oxytocin for the prevention of PPH after vaginal birth, based on newly collected evidence. PPH affects approximately 5% of births worldwide, which highlights its significance as a global health issue. It is emphasised that nearly 25% of maternal deaths are attributable to haemorrhage, making it the leading cause of maternal mortality in developing countries. The primary cause of peripartum haemorrhage is uterine atony [13]. Despite the guidelines being established several years ago, the Polish healthcare system, in the authors’ opinion, has not adequately responded to these recommendations. WHO recommends administering 10 IU of oxytocin to each patient after delivery, either intramuscularly or intravenously, as a preventive measure against PPH [14].

The authors’ view on the lack of adaptation of the healthcare system to the WHO recommendations stems, among other things, from the fact that in Poland, paramedic teams typically do not have access to oxytocin or comparable medications, as administering these drugs necessitates prior authorisation from a physician. This requirement hinders paramedics’ capacity to respond promptly and effectively in emergencies. This may be due to unfounded concerns about equipping emergency teams with oxytocin and potential concerns about side effects, as well as a need for sufficient collaboration between the obstetrician community and emergency physicians and paramedics. Additionally, if it is available, its administration requires prior authorisation from a physician, which limits the teams’ ability to respond promptly and effectively in emergencies. 

Oxytocin was the first peptide hormone to be biochemically characterised and synthesised. This relatively simple molecule, composed of nine amino acids, has been described as the ‘best-understood neuropeptide’ [15]. A growing body of evidence highlights oxytocin’s diverse functions and significant health benefits. Oxytocin has been investigated as a potential treatment for a wide range of conditions, including autism spectrum disorders, schizophrenia, and post-traumatic stress disorder, among others [16,17,18,19]. The utilisation of oxytocin in obstetrics commenced in the early 20th century. The contractile effects of oxytocin on myometrial smooth muscle were first elucidated in 1906, with its initial clinical applications focusing on managing postpartum haemorrhage. Subsequently, oxytocin gained prominence for its role in labour induction [20].

Based on the analysis conducted by Strózik et al. [21] in their 2024 study, which reported at least 879 births over a four-year period in which paramedics directly assisted with delivery, one can conclude that the percentage of preventive oxytocin use during the third stage of labour is alarmingly low. The data show that this proportion is certainly lower than 62 out of 879, equating to less than 7%. The authors regard this figure as particularly concerning.

The results achieved in Poland should be thoroughly discussed, and a corrective program addressing this situation should be implemented. In the study of Schultz et al., out-of-hospital births attended by the Queensland Ambulance Service between 1 January 2018 and 31 December 2018 were analysed. Among the 350 out-of-hospital births, oxytocin was administered after 222 deliveries, representing 63.4%. A total of 67 patients (19.1%) declined the administration of oxytocin, opting for a physiological third stage of labour. Most importantly, no adverse effects were observed in cases where the drug was administered. The median duration of the third stage of labour, defined as the time required for placental delivery, was 10 min [22].

Cash et al. [23] analysed EMS records to evaluate the management of out-of-hospital deliveries and high-risk complications by EMS teams in the US. They relied on an investigation of more than 56 million EMS calls from 2018 to 2019. They identified 3515 cases of EMS-assisted field delivery and reported oxytocin use in a small percentage (0.4%).

Our study also examined the time required for the emergency medical team to reach the patient from the moment of the call. In many developed countries, response time is a key performance indicator of the healthcare system’s efficiency. There is no doubt that unnecessary calls to pregnant women do occur, such as in cases of the premature rupture of membranes without labour contractions, where the patient could safely travel to the hospital using her transportation. However, this situation changes drastically in emergencies like cord prolapse, haemorrhage, or shoulder dystocia during home births.

Our study compared the response times of P-type and S-type emergency medical teams. Our findings appear significant, mainly due to the urgency of the events and the often critical need for an immediate response. S-type ambulances, equipped with more advanced equipment and including a physician on board, may sometimes have longer response times due to their limited availability compared to P-type ambulances, which are more common and staffed by paramedics. This highlights the crucial role of paramedics in managing prehospital childbirths. There is currently an ongoing debate in Poland about the role of emergency physicians in the prehospital setting. The constantly decreasing number of emergency physicians working in emergency medical teams is changing the nature of the ambulance subdivision. Currently, most specialised (S) teams do not include a physician. A physician is always on board the HEMS unit and in selected S teams.

Poland has a problem with a steadily declining number of births, leading to an inevitable reduction in the hospitals where maternity wards are available. This naturally increases the likelihood of emergency teams assisting women at various stages of labour and after normal and postpartum deliveries, including those with postpartum bleeding. In this context, the analysis of the use of oxytocin at the prehospital stage and the consideration of the possibility of equipping emergency medical teams with oxytocin is, in the opinion of the authors, justified. The above problems relate to Poland and several other developed countries where the rate of births is decreasing, which must cause long-term organisational changes, including those related to the EMS transportation of pregnant patients to specialised centres. 

Nehme et al. [24] identified several factors influencing the response time of EMS teams. The key independent factors included the distance to the scene, activation time, turnout time, case upgrade, time of day, day of the week, workload in the previous hour, ambulance skill set, priority zero cases (e.g., suspected cardiac or respiratory arrest), and average hospital delay time in the previous hour. Additionally, patient-dependent factors such as age, gender, chief medical complaint, and severity of the complaint were significantly associated with the emergency response time.

This study has specific limitations due to the nature of the analysis. It was based on a retrospective analysis of the medical documentation of EMS teams covering actions at the scene and during patient transport to the hospital. The data were entered into an electronic chart of EMS teams that is not specifically focused on documenting the management of pregnant or postpartum patients. This may result in documentation deficiencies regarding specific treatment in pregnant patients. Not all intervention descriptions contained sufficient data, including important information such as the Apgar score of newborns or data directly related to pregnancy. As a result, we were unable to gather as much data and expand the study to include other aspects that could have been equally interesting for exploration.

## 5. Conclusions

Oxytocin has significant applications worldwide, particularly in reducing maternal mortality associated with postpartum hemorrhage. Oxytocin is rarely administered by Polish EMS teams at the prehospital stage. Despite its well-documented effectiveness, the Polish healthcare system currently lacks a standardized protocol that requires its use by emergency responders. Incorporating oxytocin into the list of medications approved for use in prehospital care should be considered a priority. There is a need to train emergency medical personnel in the management of pregnancy-related emergencies in accordance with the current medical guidelines.

## Figures and Tables

**Table 1:** General characteristics of the study group.

Patient Age, Years (SD)	29.48 (6.25)
Number of pregnancies *n* (%)	
1st	3 (4.84)
2nd	22 (35.48)
3rd	12 (19.35)
4th	8 (12.90)
5th or more	13 (20.97)
No data	4 (6.45)
Number of labours *n* (%)	
1st	2 (3.23)
2nd	15 (24.19)
3rd	12 (19.35)
4th	6 (9.68)
5th or more	8 (12.90)
No data	19 (30.65)
Duration of pregnancy *n*(%)	
Preterm pregnancy	7 (11.29)
Term pregnancy	37 (59.68)
No data	36 (58.06)
Apgar score *n*(%)	
10 points	21 (33.87)
8 points	2 (3.23)
0 points	2 (3.23)
No data	36 (58.06)
Ongoing delivery	1 (1.61%)

**Table 2:** Time needed by the Emergency Medical Services teams to reach the patient.

Variable	N	$\bar{x}$ ± SD	Q2 (Q1–Q3)
Time, minutes	62	16.63 ± 17.37	12.25 (7.27–18.60)

Q2 (Q1–Q3), median (quartile 1–quartile 3). N—number of observations, $\bar{x}$ ± SD—mean plus/minus standard deviation, Q2 (Q1–Q3)—median (quartile 1–quartile 3).

**Table 3:** Comparison of the arrival time of particular Emergency Medical Services team types.

	Ambulance Type			Statistics		
Statistics	S	P	*U*	*Z*	*p*	*r_g_*
N, Q2 (Q1–Q3)	51, 14.60 (9.37–18.72)	11, 6.53 (5.27–18.03)	168.00	2.06	0.038	0.60

N—number of observations, Q2 (Q1–Q3)—median (quartile 1–quartile 3), S—specialized ambulance, P—basic ambulance, *U*—value U of the U–Mann–Whitney test statistics, *Z*—value Z of the U–Mann–Whitney statistics, *p—*level of statistical significance, *r_g_*—the effect size of the M-W test (the non-directional formula used for rank–dual correlation was proposed by Wendt and only takes positive values). The level of statistical significance was assumed to be α = 0.05.

## Data Availability

The Polish Ministry of Health holds administrative control and authority over the data obtained from the Central System for EMS Missions Monitoring, forming this study’s foundation. The Ministry provided the clinical data for this study after an individual institutional request to access the database.

## References

[1] Borovac-Pinheiro A., Pacagnella R., Cecatti J., Miller S., El Ayadi A., Souza J., Durocher J., Blumenthal P., Winikoff B. (2018). Postpartum hemorrhage: New insights for definition and diagnosis. Am. J. Obstet. Gynecol..

[2] Jaffer D., Singh P.M., Aslam A., Cahill A.G., Palanisamy A., Monks D.T. (2022). Preventing postpartum hemorrhage after cesarean delivery: A network meta-analysis of available pharmacologic agents. Am. J. Obstet. Gynecol..

[3] UNFPA, UNICEF, World Health Organization, World Bank Group, United Nations Population Division (2019). Trends in Maternal Mortality: 2000 to 2017|UNFPA—United Nations Population Fund. WHO, UNICEF, UNFPA, World Bank Group and the United Nations Population Division. https://www.unfpa.org/featured-publication/trends-maternal-mortality-2000-2017.

[4] Giouleka S.M., Tsakiridis I., Kalogiannidis I., Mamopoulos A., Tentas I.M., Athanasiadis A., Dagklis T. (2022). Postpartum Hemorrhage: A Comprehensive Review of Guidelines. Obstet. Gynecol. Surv..

[5] McLintock C. (2020). Prevention and treatment of postpartum hemorrhage: Focus on hematological aspects of management. Hematology.

[6] Morau E., Ducloy J., Le Roux S., Weber P., Dreyfus M. (2017). Mortalité maternelle par hémorragie, résultats de l’ENCMM, France 2010–2012. Gynecol. Obstet. Fertil. Senol..

[7] Sobhy S., Arroyo-Manzano D., Murugesu N., Karthikeyan G., Kumar V., Kaur I., Fernandez E., Gundabattula S.R., Betran A.P., Khan K. (2019). Maternal and perinatal mortality and complications associated with caesarean section in low-income and middle-income countries: A systematic review and meta-analysis. Lancet.

[8] Saucedo M., Deneux-Tharaux C., Bouvier-Colle M.-H. (2013). Ten years of confidential inquiries into maternal deaths in France, 1998–2007. Obstet. Gynecol..

[9] Leal R., Lança F. (2023). Comparison of European recommendations about patient blood management in postpartum haemorrhage. Transfus. Med..

[10] Hofer S., Blaha J., Collins P.W., Ducloy-Bouthors A.-S., Guasch E., Labate F., Lança F., Nyfløt L.T., Steiner K., Van de Velde M. (2023). Haemostatic support in postpartum haemorrhage: A review of the literature and expert opinion. Eur. J. Anaesthesiol..

[11] Diaz V., Abalos E., Carroli G. (2018). Methods for blood loss estimation after vaginal birth. Cochrane Database Syst. Rev..

[12] Executive Summary—WHO Recommendation on Routes of Oxytocin Administration for the Prevention of Postpartum Haemorrhage After Vaginal Birth—NCBI Bookshelf. https://www.ncbi.nlm.nih.gov/books/NBK564758/.

[13] Braddick L., Tuckey V., Abbas Z., Lissauer D., Ismail K., Manaseki-Holland S., Ditai J., Stokes T. (2016). A mixed-methods study of barriers and facilitators to the implementation of postpartum hemorrhage guidelines in Uganda. Int. J. Gynecol. Obstet..

[14] WHO Recommendation on Routes of Oxytocin Administration for the Prevention of Postpartum Haemorrhage After Vaginal Birth. https://www.who.int/publications/i/item/9789240013926.

[15] Jurek B., Neumann I.D. (2018). The Oxytocin Receptor: From Intracellular Signaling to Behavior. Physiol. Rev..

[16] Hurlemann R., Grinevich V. (2018). Behavioral Pharmacology of Neuropeptides: Oxytocin. https://link.springer.com/content/pdf/10.1007/978-3-319-63739-6.pdf.

[17] Goh K.K., Chen C.-H., Lane H.-Y. (2021). Oxytocin in Schizophrenia: Pathophysiology and Implications for Future Treatment. Int. J. Mol. Sci..

[18] Zhang S., Zhang Y.-D., Shi D.-D., Wang Z. (2023). Therapeutic uses of oxytocin in stress-related neuropsychiatric disorders. Cell Biosci..

[19] Persico A.M., Ricciardello A., Lamberti M., Turriziani L., Cucinotta F., Brogna C., Vitiello B., Arango C. (2021). The pediatric psychopharmacology of autism spectrum disorder: A systematic review—Part I: The past and the present. Prog. Neuro-Psychopharmacol. Biol. Psychiatry.

[20] Dale H.H. (1906). On some physiological actions of ergot. J. Physiol..

[21] Strózik M., Wiciak H., Raczyński A., Smereka J. (2025). Emergency medical team interventions in Poland during out-of-hospital deliveries: A retrospective analysis [published online as ahead of print on March 20, 2024]. Adv. Clin. Exp. Med..

[22] Schultz B.V., Hall S., Parker L., Rashford S., Bosley E. (2021). Epidemiology of Oxytocin Administration in Out-of-Hospital Births Attended by Paramedics. Prehosp. Emerg. Care.

[23] Cash R.E., Kaimal A.J., Samuels-Kalow M.E., Boggs K.M., Swanton M.F., Camargo C.A. (2024). Epidemiology of Emergency Medical Services-Attended out-of-Hospital Deliveries and Complications in the United States. Prehosp. Emerg. Care.

[24] Nehme Z., Andrew E., Smith K. (2016). Factors Influencing the Timeliness of Emergency Medical Service Response to Time Critical Emergencies. Prehosp. Emerg. Care.

